# Aging-Related Alterations of Glymphatic Transport in Rat: *In vivo* Magnetic Resonance Imaging and Kinetic Study

**DOI:** 10.3389/fnagi.2022.841798

**Published:** 2022-03-10

**Authors:** Lian Li, Guangliang Ding, Li Zhang, Esmaeil Davoodi-Bojd, Michael Chopp, Qingjiang Li, Zheng Gang Zhang, Quan Jiang

**Affiliations:** ^1^Department of Neurology, Henry Ford Health System, Detroit, MI, United States; ^2^Department of Physics, Oakland University, Rochester, MI, United States

**Keywords:** glymphatic clearance, aged brain, DCE-MRI, advanced kinetic model, olfactory bulb

## Abstract

**Objective:**

Impaired glymphatic waste clearance function during brain aging leads to the accumulation of metabolic waste and neurotoxic proteins (e.g., amyloid-β, tau) which contribute to neurological disorders. However, how the age-related glymphatic dysfunction exerts its effects on different cerebral regions and affects brain waste clearance remain unclear.

**Methods:**

We investigated alterations of glymphatic transport in the aged rat brain using dynamic contrast-enhanced magnetic resonance imaging (DCE-MRI) and advanced kinetic modeling. Healthy young (3–4 months) and aged (18–20 months) male rats (*n* = 12/group) underwent the identical MRI protocol, including T2-weighted imaging and 3D T1-weighted imaging with intracisternal administration of contrast agent (Gd-DTPA). Model-derived parameters of infusion rate and clearance rate, characterizing the kinetics of cerebrospinal fluid (CSF) tracer transport via the glymphatic system, were evaluated in multiple representative brain regions. Changes in the CSF-filled cerebral ventricles were measured using contrast-induced time signal curves (TSCs) in conjunction with structural imaging.

**Results:**

Compared to the young brain, an overall impairment of glymphatic transport function was detected in the aged brain, evidenced by the decrease in both infusion and clearance rates throughout the brain. Enlarged ventricles in parallel with reduced efficiency in CSF transport through the ventricular regions were present in the aged brain. While the age-related glymphatic dysfunction was widespread, our kinetic quantification demonstrated that its impact differed considerably among cerebral regions with the most severe effect found in olfactory bulb, indicating the heterogeneous and regional preferential alterations of glymphatic function.

**Conclusion:**

The robust suppression of glymphatic activity in the olfactory bulb, which serves as one of major efflux routes for brain waste clearance, may underlie, in part, age-related neurodegenerative diseases associated with neurotoxic substance accumulation. Our data provide new insight into the cerebral regional vulnerability to brain functional change with aging.

## Introduction

Aging is accompanied by a wide array of progressive and deteriorating changes in the brain (Peters, 2006; Kirkwood, 2010; Harada et al., 2013). These broad-spectrum and complex changes occur at all levels (e.g., from molecules to morphology), profoundly impacting the structure and function of the brain and cognition. Along with these gradual and subtle changes that deleteriously affect the brain, undesirable alterations with age are present in the glymphatic transport pathway (Benveniste et al., 2019b; Zhang et al., 2019), a perivascular network involving cerebrospinal fluid (CSF) recirculation throughout the brain and interstitial solute clearance from the central nervous system (CNS) (Jessen et al., 2015; Plog and Nedergaard, 2018). Studies of the glymphatic system show that a large proportion of subarachnoid CSF reenters the brain parenchyma along peri-arterial spaces, exchanges with the interstitial fluid (ISF) and exits the brain along peri-venous spaces (Iliff et al., 2012, 2013). This process, supported by astrocytic aquaporin-4 (AQP4) water channels, facilitates removal of metabolic waste products (Jessen et al., 2015; Mestre et al., 2018). Age-related cognitive decline and neurodegenerative disorders are associated with the misaggregation of proteins (e.g., amyloid-β, tau) in the brain (Yankner et al., 2008; Mawuenyega et al., 2010; Peng et al., 2016), attributed in part to the compromised glymphatic clearance function that occurs with advancing age (Jessen et al., 2015; Boland et al., 2018; Benveniste et al., 2019b). However, how the age-related glymphatic dysfunction exerts its effects on different cerebral regions and affects brain waste clearance remain unclear.

To ascertain solute transport via the glymphatic system, imaging modalities with CSF tracers are employed to visualize and monitor the surrogate “waste” solutes as they pass through the brain (Benveniste et al., 2019b). Compared to two-photon imaging and fluorescence microscopy which offer either limited field of view or cross-sectional evaluations (Plog and Nedergaard, 2018), magnetic resonance imaging (MRI) captures the whole brain and provides dynamic measurements in a non-invasive way, spatiotemporally appropriate for the investigation of brain-wide glymphatic pathway function (Iliff et al., 2013; Ratner et al., 2017). As a clinically relevant technique, dynamic contrast-enhanced MRI (DCE-MRI) has been used to track the trajectory of CSF tracer via the glymphatic system as well as to model the kinetic features of glymphatic transport in the live brain (Iliff et al., 2013; Jiang et al., 2017; Ratner et al., 2017; Davoodi-Bojd et al., 2019). Based on the time series of contrast-induced signal changes on 3D T1-weighted images, we developed a two-compartment mathematical model (Davoodi-Bojd et al., 2019). By using the local input function (IF), the errors arising from the global IF (Lee et al., 2015) were largely reduced. With this advantage, the kinetics of glymphatic transport represented by CSF tracer movement in the brain can then be more accurately estimated using the model-derived parameters. This model, for the first time, demonstrates an improved capacity of DCE-MRI measures of glymphatic transport to differentiate between diseased animals (e.g., diabetes) and healthy controls (Jiang et al., 2017; Davoodi-Bojd et al., 2019). Thus, use of DCE-MRI in conjunction with our advanced modeling would reveal more detailed information regarding the alterations of glymphatic transport function in the aging brain.

Recent studies show that glymphatic function in the aged brain is disrupted, as reflected by the lessened CSF penetration, reduced CSF and ISF exchange, and insufficient waste elimination (Hawkes et al., 2011; Kress et al., 2014). As revealed at gene (Berchtold et al., 2008; Murugesan et al., 2012), neuronal (Mattson and Magnus, 2006; Mora et al., 2007), microvascular (Murugesan et al., 2012), and anatomic (Mora et al., 2007) levels, aging of the brain is characterized by heterogeneous, asynchronous, and region-specific changes with time. Emerging data also indicate that certain brain regions appear more prone to pathophysiological consequences (e.g., injury and/or stress) with increasing age compared with other brain regions (Mattson and Magnus, 2006; Fjell et al., 2014; Feng et al., 2020). Despite evidence linking age with disrupted glymphatic activity (Hawkes et al., 2011; Kress et al., 2014; Zhou et al., 2020), how the compromised glymphatic function affects various anatomical regions in the aged brain and whether there exist cerebral regions vulnerable to age-related impairment of glymphatic function remain to be elucidated. Clarification of these issues may expand our knowledge of the normal aging process and reveal potential mechanisms underlying age-related disorders, and the objective measures of glymphatic kinetics in the young and aged brain may also provide insights into the regional vulnerability to glymphatic dysfunction during brain aging.

With DCE-MRI and our advanced modeling, the current study was designed to detect the alterations of glymphatic function in the aged brain. The kinetic changes in glymphatic transport, characterized by model-derived parameters, were investigated in discrete and representative brain regions of young vs. aged male rat with a focus on both influx and efflux features. Our data reveal that a severe suppression of glymphatic activity occurs in the olfactory bulb of the aged male brain, substantially hindering the solute drainage via the olfactory efflux route and largely accounting for the disrupted brain waste clearance.

## Materials and Methods

All experimental procedures were approved by the Institutional Animal Care and Use Committee of Henry Ford Health System and carried out in accordance with the NIH Guide for the Care and Use of Laboratory Animals.

### Animals and Experimental Procedures

Male Wistar rats (Charles River, Wilmington, MA, US) were used in the present study. Adult (3–4 months, ∼ 400 g, *n* = 12) and aged (18–20 months, ∼ 600 g, *n* = 12) rats were subjected to the identical experimental procedures, including the surgical preparation for contrast administration via the cisterna magna, and subsequent MRI measurements.

Catheter implantation surgery was performed prior to performing MRI scans (Ding et al., 2018). Briefly, the rats were initially anesthetized by inhalation of 3% isoflurane and maintained in the range of 1.0–1.5% isoflurane in a mixture of N_2_O (70%) and O_2_ (30%) via a nose mask throughout the surgical period. Rectal temperature was strictly controlled at 37°C ± 1°C using a feedback-regulated water heating system. The head of the anesthetized rat was mounted in a stereotactic frame with care to permit spontaneous breathing. After the atlanto-occipital membrane was exposed using a midline dorsal neck incision, a polyethylene catheter (PE-10 tubing; Becton Dickinson, MD, United States) filled with saline was inserted into the subarachnoid cisterna magna space via a small durotomy made with a 27 gauge needle. The outside part of catheter was fixed onto the occipital bone with superglue and the skin incision was closed around the catheter.

MR imaging was performed with a 7T system (Bruker–Biospin, Billerica, MA, United States) (Ding et al., 2018). A birdcage type coil was used as the transmitter and a quadrature half-volume coil as the receiver. The animal with catheter implantation was securely fixed on a MR-compatible holder equipped with an adjustable nose cone for administration of anesthetic gases and stereotaxic ear bars to immobilize the head. For reproducible positioning of the animal in the magnet, a fast-gradient echo imaging sequence was used at the beginning of each MRI session. During image acquisition, anesthesia was maintained by a gas mixture of N_2_O (70%) and O_2_ (30%) with 1.0–1.5% isoflurane (Piramal Inc., Bethlehem, PA, United States), and rectal temperature was kept at 37 ± 1^°^C using a feedback controlled air heating blower (Rapid Electric, Brewster, NY, United States).

To detect the structural changes, T2-weighted imaging (T2WI) (TE = 8, 16, 24, 32, 40, 48, 56, 64, 72 and 80 ms, TR = 4 s, FOV = 32 × 32 mm^2^, matrix = 128 × 128, 13 slices, thickness = 1 mm) was measured. To monitor the dynamic influx and clean-out process, 3D T1-weighted imaging (T1WI) (TE = 4 ms, TR = 18 ms, flip angle = 12°, FOV = 32 × 32 × 16 mm^3^, matrix = 256 × 192 × 96) with contrast agent of Gd-DTPA was acquired. The time series of T1WI scanning continued for 6 h, starting with three baseline scans followed by intra-cisterna magna Gd-DTPA (21 mM concentration) delivery at a constant infusion rate of 1.6 μl/min over 50 min (Jiang et al., 2017; Ding et al., 2018) via the indwelling catheter connected a 100 μl syringe (Hamilton Robotics, Reno, NV, US) mounted on an infusion pump (Harvard Apparatus, Holliston, MA, US).

### Magnetic Resonance Imaging Data Processing

**FIGURE 1:**
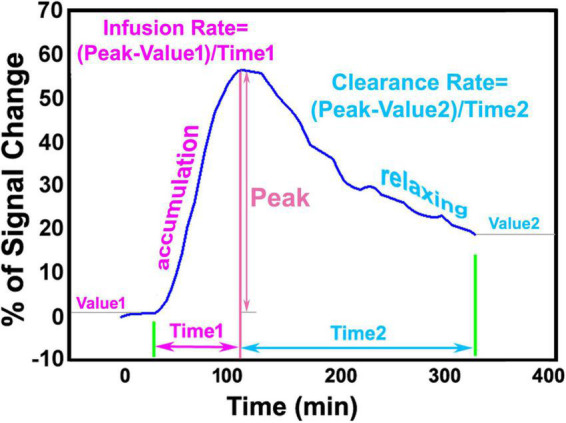
Representative time signal curve (TSC) of a tissue cluster, illustrating infusion rate and clearance rate obtained from accumulation phase and relaxing phase, respectively.

The step-by-step procedures for DCE-MRI data processing and parametric map generation have been previously described (Davoodi-Bojd et al., 2019). To correct for motion that occurs during the 6-h scan, the entire set of sequential images for each animal were co-registered to its initial volume. Then, 3D T1WIs for all animals were co-registered to a standard reference template so that the comparison between groups will be carried out in the common spatial space. With the changes of MRI signal that correspond to the time trajectories of CSF tracer concentrations, brain voxels were clustered into similar regions based on their dynamic responses to the infusion of contrast agent. Time signal curve (TSC) for each cluster that represents the retention of infused tracer as a function of time in the tissue region was obtained, yielding the required information for our advanced kinetic modeling. Using a defined approach with specific criteria, a local input function for any formed cluster was found among the TSCs of its neighboring clusters, largely reducing errors arising from the global TSC of the whole brain (Lee et al., 2015). For each tissue cluster, the parameters characterizing the kinetics of tracer uptake and clearance were derived from its own TSC. Herein, infusion rate is defined by the rate of signal increase from the point immediately after three baseline scans to the peak in the accumulation phase of the TSC, while clearance rate is defined by the rate of signal decrease from the peak to the end of experiment in the relaxing phase of the TSC (Figure 1). After calculating these kinetic parameters in each cluster from its average TSC, parametric maps of infusion rate and clearance rate for whole brain were then generated.

### Quantification and Statistical Analysis

**FIGURE 2:**
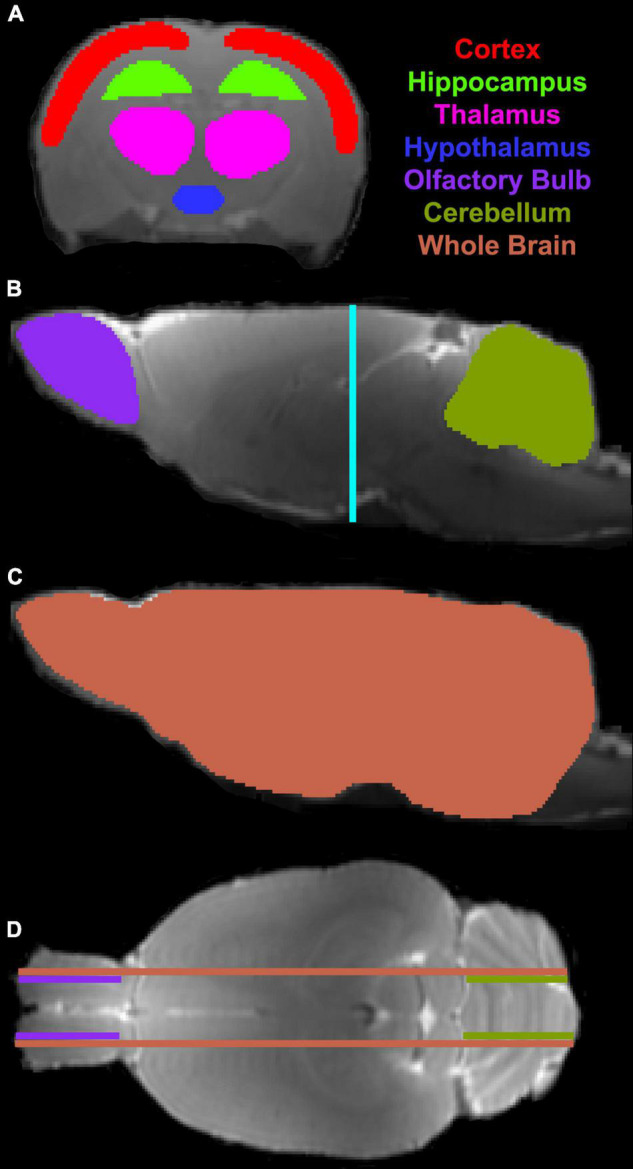
Regions of interest (ROIs) (colored anatomical areas in **A–C**) on the coronal (**A**, Bregma -3.00 mm) and sagittal (**B,C**, Lateral 0.40 mm) sections, and their corresponding locations on the sagittal (**B**, light blue line) and axial (**D**, purple, olive and coral lines, Interaural 5.72 mm) sections, respectively.

On T2 map, ventricular areas were specified by those pixels with a T2 value higher than the mean plus twice the standard deviation (mean + 2 *SD*) provided by the surrounding tissue (Li et al., 2011). The representative structural locations for the third (3V), fourth (4V) and lateral (LV) ventricles were determined, and all animals were, respectively, estimated in these same locations. For each ventricle, ventricular size was identified on three contiguous coronal slices at the corresponding structural location, and its volume was then obtained by adding all the areas measured on individual slices and multiplying the total by the slice thickness. To evaluate the kinetic features of contrast agent transport via the glymphatic system within the brain, regions of interest (ROIs) encompassing representative brain tissue areas (including cortex, hippocampus, thalamus, hypothalamus, olfactory bulb and cerebellum) were created on the fixed coronal and sagittal sections of 3D T1WI (Figure 2). With these ROIs, measurements were conducted on the parametric maps and averaged in each ROI for different groups. Results are presented as mean ± standard error. To detect the aging effects on glymphatic transport function characterized by the kinetic parameters in these distinct brain regions and on structural change reflected by ventricular enlargement, a two-sample *t*-test was performed with *p* < 0.05 inferred for statistical significance.

## Results

### Contrast Uptake Pattern in the Young and Aged Brain

**FIGURE 3:**
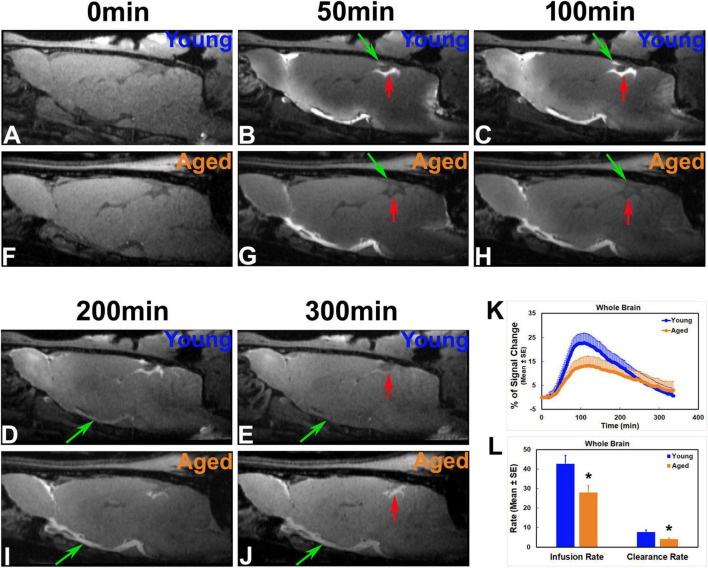
Progressive transport of intracisterna-injected contrast agent in the representative young **(A–E)** and aged **(F–J)** rat brain captured by T1WIs (**A–J**, Lateral 0.40 mm), group TSCs **(K)** and parametric quantification **(L)**. Compared to the young brain, later signal appearance (**B** vs. **G**; **C** vs. **H** at the same areas indicated by green and red arrows, respectively), lower magnitude and narrower extent in tissue enhancement (**B** vs. **G**; **C** vs. **H** in the tissue areas of olfactory bulb and along the base of the brain) and slower clean-out of administered contrast (**D** vs. **I**; **E** vs. **J** at the same areas indicated by green and red arrows, respectively) were observed in the aged brain. Reduced efficiency in both glymphatic influx and efflux was detected in the aged animals than in the young animals as evaluated by TSCs **(K)** as well as kinetic parameters of infusion and clearance rates **(L)**. *p < 0.05 (Young vs. Aged).

**FIGURE 4:**
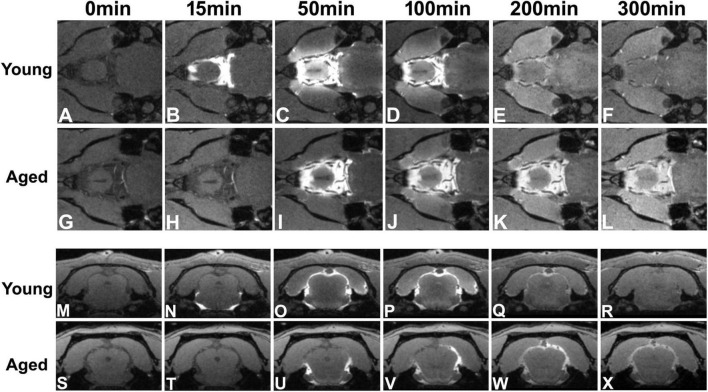
Flow of contrast at the level of circle of Willis **(A–L)** and the level of pineal gland (**M–X**, Bregma −7.92 mm) in the representative young and aged rat brain (the same animals shown in Figure 3). Earlier arrival (**B** vs. **H**) and shorter retention period (from **B–D** vs. from **I–L**) of contrast at the level of circle of Willis were present in the young brain than in the aged brain. With time after intracisternal injection, the movement of contrast from the base of the brain toward the pineal gland was captured in both young (comparing **N** with **O**) and aged (comparing **U** with **V,W**) brain. However, much slower progression (from **N–O** vs. from **U–W**) and more sluggish clear-out (**R:** almost clean vs. **X:** still retained) of contrast over time were found in the aged brain than in the young brain.

Depicted by temporal and spatial progression of contrast uptake in the brain following intracisternal administration, our DCE-MRI captured a glymphatic transport pattern that was visually and macroscopically similar in both young and aged brain (Figures 3, 4). Regardless of age, apparent contrast signals appeared at early times (∼ 10 min post-injection) along the base of the brain. When gradually expanding afterward in the tissue along the ventral surface of the brain, the contrast moved up toward the areas of olfactory bulb and pineal gland where a more rapid increase in signal intensity was manifest compared to other tissue regions (Figure 3). As the key CSF transport pathways (Iliff et al., 2013; Ratner et al., 2017), this contrast flow pattern in the live rodent brain visualizes how the solute moves from the subarachnoid space of the cisterna magna into the brain parenchyma via the glymphatic system. Our imaging data demonstrated that this typical glymphatic transport pattern was present in both the young and aged brain.

Contrast-induced enhancement was stronger in the olfactory bulb, hypothalamus and cerebellum than in the cortex, hippocampus and thalamus in both young and aged brain. Compared to the anatomical regions distal from the transport pathways, more pronounced enhancement was detected in the tissue regions immediately associated with the glymphatic transport pathways (e.g., olfactory bulb) or immediately adjacent to the glymphatic influx nodes of pituitary and pineal gland recesses (Iliff et al., 2013; Ratner et al., 2017) (e.g., hypothalamus).

### Changes of Glymphatic Transport in the Aged Brain Visualized by Dynamic Contrast-Enhanced Magnetic Resonance Imaging

Figures 3, 4 show the progressive glymphatic transport of contrast agent from the CSF reservoir of the cisterna magna into brain parenchyma in representative young and aged rat brain. As revealed on the sagittal section (Figure 3), later signal appearance (Figure 3B vs. Figure 3G and Figure 3C vs. Figure 3H in the same areas indicated by green and red arrows, respectively), lower magnitude and narrower extent in tissue enhancement (Figure 3B vs. Figure 3G and Figure 3C vs. Figure 3H in the tissue areas of olfactory bulb and along the base of the brain), and slower clean-out of administrated contrast (Figure 3D vs. Figure 3I and Figure 3E vs. Figure 3J in the same areas indicated by green and red arrows, respectively) were observed in the aged brain compared to the young brain. The transport differences were present at the level of circle of Willis (Figures 4A–L), evidenced by earlier arrival (Figure 4B vs. Figure 4H) and shorter retention period (from Figures 4B–D vs. from Figures 4I–L) of contrast in the young brain than in the aged brain. Our dynamic images captured the movement of contrast from the base of the brain toward olfactory bulb (Figure 3) and pineal gland (Figures 4M–X) in both young and aged brain. However, much slower progression (Figure 3B vs. Figure 3G in olfactory bulb) and more sluggish clearance of contrast (Figure 4R: almost clean vs. Figure 4X: still retained in pineal gland) over time were found in the aged brain than in the young brain.

### Changes of Cerebrospinal Fluid-Filled Compartments in the Aged Brain

**FIGURE 5:**
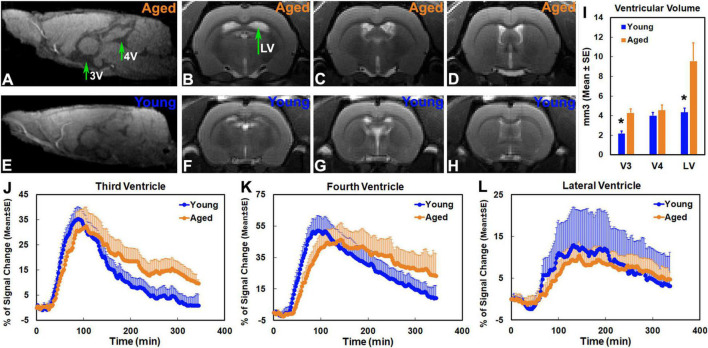
Sagittal section (**A,E**, Lateral −0.10 mm) at the level of the third (3V) and fourth (4V) ventricles, coronal sections (**B–D,F–H**, Bregma −1.72 mm, −0.72 mm, 0.24 mm) at the representative structural locations containing the lateral ventricles (LV), ventricular volumes **(I)** and group TSCs **(J–L)** obtained from the corresponding ventricles. Ventricular enlargement was present in the aged brain **(A–D** vs. **E–H)**. Significantly expanded third and lateral ventricles were detected in the aged brain as compared to the young brain (**I**, *p < 0.03, Young vs. Aged in the same ventricle). Compared to the young brain, a reduced pace for contrast movement through the third **(J)** and fourth **(K)** ventricles was detected in the aged brain. In addition to the decreased maximum levels of TSCs **(J,K)**, prolonged periods of time were taken in the aged brain to attain the peak values of TSCs and to attenuate the signal intensities afterward **(J,K)**. Nevertheless, reduced differences in group TSCs between young and aged brain were found in lateral ventricles **(L)**.

As demonstrated at representative structural locations on the sagittal and coronal sections that contained the third, fourth and lateral ventricles (Figure 5), notable ventricular enlargement was found in the aged brain (Figures 5A–D vs. Figures 5E–H). Significantly expanded third and lateral ventricles were detected in the aged brain as compared to the young brain (Figure 5I). Meanwhile, a reduced pace for CSF flow through the ventricles, characterized by contrast-induced TSCs, was present in the aged brain. Compared to the young brain in the third (Figure 5J) and fourth (Figure 5K) ventricles, prolonged periods of time were taken in the aged brain to attain the peak values of TSCs (**3V**: 108.46 ± 3.44 min vs. 84.83 ± 2.78 min, *p* < 0.001; **4V**: 129.71 ± 10.86 min vs. 94.00 ± 6.45 min, *p* < 0.009) and to attenuate the signal intensities afterward (**3V**: 9.65 ± 1.48 vs. 0.80 ± 4.26, % of signal change at the end of experiment, *p* < 0.04), indicating a reduced efficiency in influx and clearance of contrast agent in these CSF-filled regions. These kinetic differences between two groups, nevertheless, were less evident in the lateral ventricles (Figure 5L).

### Changes of Glymphatic Transport in the Aged Brain Characterized by Advanced Kinetic Modeling

**FIGURE 6:**
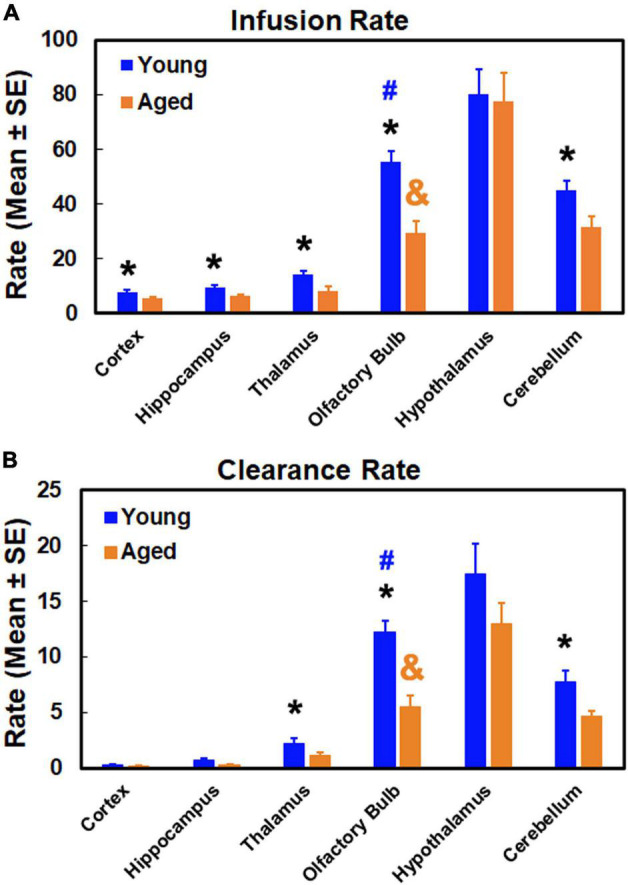
Quantitative results of infusion rate **(A)** and clearance rate **(B)** in examined anatomical regions. *p < 0.05, Young vs. Aged in the same region; ^#^*p* < 0.05, olfactory bulb vs. the other regions in the young group; ^&^*p* < 0.05, olfactory bulb vs. the other regions in the aged group except the cerebellum.

Glymphatic transport kinetics was evaluated in the representative anatomical regions with model-derived parameters of infusion rate and clearance rate (Figure 6). Among the examined regions, our parametric quantification showed that infusion rate and clearance rate are higher in olfactory bulb, hypothalamus and cerebellum than in cortex, hippocampus and thalamus in both young and aged brain, indicating the regional differences in glymphatic transport. Aged brain exhibited reduced infusion and clearance rates across the brain relative to their young counterparts, with significant decline in infusion rate detected in all measured regions except in the hypothalamus (Figure 6A), and significant decrease in clearance rate in olfactory bulb, thalamus and cerebellum (Figure 6B).

### Impact of Age-Related Glymphatic Dysfunction on Different Anatomical Regions

As summarized in Figure 6, age-related impairment of glymphatic transport function, characterized by reduced infusion (Figure 6A) and clearance (Figure 6B) rates compared to the young brain, affected each of the examined anatomical regions. While the degree of glymphatic dysfunction in the aged brain varied among the brain regions, our data demonstrated that a severe deterioration in glymphatic function occurred in the olfactory bulb, evidenced by the dramatic decline in both infusion and clearance rates. As shown in Figure 6, both kinetic parameters in the olfactory bulb decreased to such an extent in the aged brain that they remarkably changed the value pattern representing the relationship between the anatomical regions. In the young brain, infusion and clearance rates in the olfactory bulb were significantly lower than in the hypothalamus, and significantly higher than in the other examined regions. These significant differences between olfactory bulb and cerebellum for both parameters in the young brain, however, were absent in the aged brain due to considerably reduced infusion and clearance rates in olfactory bulb. With the reduced parametric values present throughout the anatomical regions compared to the young brain, the significant differences between olfactory bulb and other regions remained in the aged brain.

## Discussion

Our dynamic imaging and kinetic quantification demonstrated a widespread impairment of glymphatic transport in the aged brain, characterized by an overall reduction of both glymphatic influx and efflux compared to the young brain. While the effects of age on the glymphatic function were evident throughout the brain, a distinctive and dramatic decline in both glymphatic infusion rate and clearance rate was detected in olfactory bulb as compared with other cerebral regions, indicating the heterogeneous and regional preferential alterations of glymphatic function with aging. The severely deteriorated efficiency in solute transport via the olfactory efflux route may largely account for the compromised waste clearance from the aged brain, thereby facilitating the accumulation of neurotoxic products that contribute to age-related cognitive decline and neurodegenerative diseases.

On a series of whole-brain 3D images (T1WI), the enhancement pattern represents the distribution and concentration of contrast agent that entered, traveled and redistributed within the brain via the glymphatic system following intracisternal administration. DCE-MRI therefore enables us to track the spatiotemporal dynamics of solute transport across the brain, portrayed by contrast trajectory. Consistent with earlier reports (Iliff et al., 2013; Ratner et al., 2017), typical glymphatic transport pathways, characterized by influx nodes and penetration routes, were observed in both young and aged brain (Figures 3, 4). Meanwhile, our imaging data exhibited the regional differences in the magnitude of contrast uptake into the brain parenchyma. In addition to the heterogeneity in expression pattern of AQP4 (Hoddevik et al., 2017) that plays an essential role in glymphatic fluxes (Iliff et al., 2012; Jessen et al., 2015), these variations in contrast uptake among the cerebral tissue regions appeared, at least in part, related to the distinct transport pathways that were closely associated with large arteries and arterial complex (Iliff et al., 2013; Ratner et al., 2017). Importantly, notable alterations in solute transport in the aged brain, evidenced by slower influx speed and longer efflux period compared to the young brain, were dynamically revealed on DCE-MRI (Figures 3, 4). These age-related alterations in solute transport were present in the brain tissue areas as well as in the CSF-filled compartments (Figure 5), suggesting a broad change in CSF flow dynamics. The decline in CSF pressure (Fleischman et al., 2012) and decrease in CSF production and turnover with aging (Preston, 2001; Serot et al., 2003; Chiu et al., 2012) may contribute to or underlie the alterations observed in these compartments. As expected, expanded ventricles (Figures 5A–I), a prominent feature of the aging brain (Scahill et al., 2003; Jack et al., 2008; Driscoll et al., 2009; Apostolova et al., 2012; Hamezah et al., 2017), were detected in the aged animals as compared to their young counterparts, supporting the premise that morphological modifications are concurrent with functional alterations with aging (Peters, 2006; Mora et al., 2007). As showed by TSCs (Figures 5J–L), the reduced signal increases found in the lateral ventricles compared to the signal increases in the third and fourth ventricles are likely due to the fact that the majority of CSF is produced within the two lateral ventricles from where CSF moves in a single outward direction (Sakka et al., 2011). The expanded lateral ventricles (Figures 5B–D vs. Figures 5F–H) and reduced CSF production with aging (Preston, 2001; Serot et al., 2003) may contribute to the lower variations of TSC in the aged brain than in the young brain (Figure 5L).

In addition to the imaging data that visually reveal glymphatic transport, our advanced modeling and model-derived parameters permit us to quantitatively evaluate the kinetics of solute transport via the glymphatic system. For all examined regions, both infusion and clearance rates were reduced in the aged brain compared to the young brain (Figure 6), indicating the age-related glymphatic dysfunction. Age-related changes in CSF flow dynamics that are associated with decreased CSF production (Preston, 2001; Serot et al., 2003; Chiu et al., 2012), increased CSF outflow resistance (May et al., 1990; Czosnyka et al., 2004), reduced lymphatic CSF transport (Nagra and Johnston, 2007; Ma et al., 2017) and dural lymphatic dysfunction (Park et al., 2020) may play an important role in the decline of both infusion and clearance rates in the aged brain. The degree of reduction in infusion rate differs from clearance rate, likely reflecting the different aging effects on glymphatic influx and efflux. The suppression of this brain-wide perivascular transport may in part be attributed to the age-dependent alterations in the cerebral vascular system, including the decline in vascular pulsatility (Kress et al., 2014; Jessen et al., 2015), increase in vessel stiffness (Kyrtsos and Baras, 2015; Benveniste et al., 2019b), loss of perivascular AQP4 polarization (Kress et al., 2014; Zeppenfeld et al., 2017), abnormalities in perivascular space (Laveskog et al., 2020; Zong et al., 2020), decrease in microvascular density (Bullitt et al., 2010; Murugesan et al., 2012; Reed et al., 2018; Watanabe et al., 2020) and neurovascular uncoupling (Venkat et al., 2016; Toth et al., 2017).

Although the aging effects on both glymphatic influx and efflux were apparent and widespread, our data demonstrated that the aged brain was associated with a distinct pattern of regional vulnerability reflected by a severe deterioration in glymphatic transport function in olfactory bulb (Figure 6). As one of the major efflux pathways (Murtha et al., 2014; Norwood et al., 2019; Brady et al., 2020), inefficient drainage through olfactory route slows down the whole brain clearance and favors the accumulation of toxic metabolites and proteins, rendering the aged brain more susceptible to neurodegenerative disorders. In support of our observations, previous studies have shown that amyloid-β deposition in olfactory bulb occurs not only earlier than in other brain regions (Wesson et al., 2010), but also prior to the appearance of cognitive symptoms (Attems et al., 2014). Importantly, glymphatic disruption precedes the presence of significant amyloid-β deposition (Peng et al., 2016). The pronouncedly reduced activity of solute transport in the olfactory bulb in the aged brain may partially reflect the effect of olfactory impairment (OI) that is prevalent in the elderly population (Mobley et al., 2014; Van Regemorter et al., 2020). The olfactory sensory nerves serve as a CSF outflow pathway (Norwood et al., 2019). Age-dependent decrease in the number of olfactory sensory neurons (Mobley et al., 2014) and decline in neurogenesis capacity (Enwere et al., 2004; Child et al., 2018), and neurodegeneration (Hussain et al., 2018; Bhatia-Dey and Heinbockel, 2021) that parallel with OI (Mobley et al., 2014) may increase the resistance to CSF flow (Albeck et al., 1998; Norwood et al., 2019), negatively affecting solute drainage through the olfactory efflux route. Olfactory deficit has been linked to advanced physiological brain aging (Devanand et al., 2015; Park et al., 2021) and has been associated with the forthcoming neurodegenerative disorders (Baba et al., 2012; Yoo et al., 2019), although the mechanism behind these associations remains to be explored. The coexistence of OI and severe suppression of glymphatic transport with advancing age may provide new insights into these connections. While OI shows an early marker of age-related cognitive decline and neurodegenerative disorders with the involvement of neurotoxic product aggregation (Denver and McClean, 2018), our data indicate that its predictive power may largely be attributed to the inefficiency in solute clearance through the olfactory drainage route where a great reduction of perivascular transport occurs (Figure 6). By influencing the waste burden of the brain, the severity of glymphatic transport dysfunction along this major efflux route seems to play an important role in driving the normal aging trajectory toward pathological degeneration. Therefore, this brain regional vulnerability to age-related glymphatic dysfunction, as revealed by our kinetic evaluation (Figure 6), may in part underlie various neurodegenerative disorders.

We are aware of limitations in the current study. First, we only used male rats for young and aged groups instead of both genders. Although this experimental design is supported by the previous finding that there is no sex-dependent difference in glymphatic influx in mice with age ranging from young (2–4 months) to old (22–24 months) (Giannetto et al., 2020), further examination in rodents for sexual dimorphism may be required, particularly for both glymphatic influx and efflux. Secondly, during the MRI scan, animals were anaesthetized with isoflurane which is one of the anesthetic regimens applicable and necessary for hours of measurements. Although controversial, anesthetic effects on glymphatic function have been detected (Gakuba et al., 2018; Benveniste et al., 2019a; Hablitz et al., 2019; Stanton et al., 2021). We are cognizant of the possibility that even under the same anesthetic regimen for all animals studied, potential disparate effects of isoflurane on young vs. aged rats may exist. Clearly, additional studies, especially including the factors of age, are needed to determine the precise role of anesthesia on the activity of glymphatic system. As a caveat of the present study, technical limitations prevented us from analyzing more specific cerebral tissue regions, such as sub-regions of the cortex and thalamus. In addition, an important caveat is the anatomical and multiple other differences between the animal and the human. Thus, while the olfactory bulb occupies a large portion of the rat brain, it accounts for a small part of the human brain, and our data cannot be simply extrapolated to the human, but, primarily provide insight into the role of distinct pathways contributing to glymphatic function in the aged brain.

In summary, DCE-MRI together with our kinetic approach revealed a unique pattern of compromised glymphatic transport function in the aged brain, represented by altered kinetic features in distinct brain regions as compared to the young brain. In addition to an overall suppression of glymphatic activity, our data further demonstrate that the magnitude of impaired glymphatic function exhibits a regional preference, and that olfactory bulb appears to be a cerebral region particularly prone to age-related glymphatic dysfunction. This age-vulnerability renders the brain more susceptible to the insults resulting from toxic waste aggregation, and therefore, strategies for maintaining efficient glymphatic clearance through olfactory efflux route may mitigate the cognitive decline with age and prolong the healthy aging.

## Data Availability Statement

The original contributions presented in the study are included in the article/supplementary material, further inquiries can be directed to the corresponding author/s.

## Ethics Statement

The animal study was reviewed and approved by the Institutional Animal Care and Use Committee of Henry Ford Health System.

## Author Contributions

LL wrote the manuscript and performed MRI data processing and analysis. GD and QL performed MRI experiments and data analysis and interpretation. LZ conducted the specific surgery for MRI experiments and data acquisition. ED-B performed data processing and modeling. MC, ZZ, and QJ contributed to conception and design of the study and manuscript revision. All authors contributed to the article and approved the submitted version.

## Conflict of Interest

The authors declare that the research was conducted in the absence of any commercial or financial relationships that could be construed as a potential conflict of interest.

## Publisher’s Note

All claims expressed in this article are solely those of the authors and do not necessarily represent those of their affiliated organizations, or those of the publisher, the editors and the reviewers. Any product that may be evaluated in this article, or claim that may be made by its manufacturer, is not guaranteed or endorsed by the publisher.
